# Sensing of Vascular Permeability in Inflamed Vessel of Live Animal

**DOI:** 10.1155/2018/5797152

**Published:** 2018-05-15

**Authors:** Sang A Park, Soi Jeong, Young Ho Choe, Young-Min Hyun

**Affiliations:** Department of Anatomy and Brain Korea 21 PLUS Project for Medical Science, Yonsei University College of Medicine, Seoul 03722, Republic of Korea

## Abstract

Increase in vascular permeability is a conclusive response in the progress of inflammation. Under controlled conditions, leukocytes are known to migrate across the vascular barriers to the sites of inflammation without severe vascular rupture. However, when inflammatory state becomes excessive, the leakage of blood components may occur and can be lethal. Basically, vascular permeability can be analyzed based on the intensity of blood outflow. To evaluate the amount and rate of leakage in live mice, we performed cremaster muscle exteriorization to visualize blood flow and neutrophil migration. Using two-photon intravital microscopy of the exteriorized cremaster muscle venules, we found that vascular barrier function is transiently and locally disrupted in the early stage of inflammatory condition induced by N-formylmethionyl-leucyl-phenylalanine (fMLP). Measurement of the concentration of intravenously (i.v.) injected Texas Red dextran inside and outside the vessels resulted in clear visualization of real-time increases in transient and local vascular permeability increase in real-time manner. We successfully demonstrated repeated leakage from a target site on a blood vessel in association with increasing severity of inflammation. Therefore, compared to other methods, two-photon intravital microscopy more accurately visualizes and quantifies vascular permeability even in a small part of blood vessels in live animals in real time.

## 1. Introduction

Control of vascular permeability allows leukocyte migration, provides nutrition, and maintains homeostasis of the body [1]. Due to its importance, therefore, vascular permeability has been actively investigated in the field of medical science [2–4]. Various stimuli act on endothelial cells and adjust the tightness of the vascular barrier by remodeling actins [3]. VE-cadherin, which is only expressed in the epithelial cell, and JAM-1 are key factors in vascular hemostasis and angiogenesis [5, 6]. Metastasis of cancer cells is strongly associated with vascular integrity since a highly permeable endothelial barrier induces angiogenesis, which eventually supports the dissemination of tumor cells [7–9]. Despite numerous studies, detailed steps of controlling mechanisms about vascular permeability have not been uncovered. Therefore, we prepared cremaster muscle of mice in order to observe the changes in vascular integrity in acute inflammatory conditions. The cremaster muscle was treated with bacterial chemoattractant, fMLP, and two-photon intravital imaging of cremaster muscle venules was conducted for more than 3 hours. fMLP was used to recruit neutrophils along the cremaster vessels [10, 11]. The major premise of this study was that neutrophils activated by fMLP would increase the secretion of vascular endothelial growth factor A (VEGF-A); therefore, vascular integrity would be decreased by cytoskeletal contraction [2, 12]. We were able to detect increased vascular permeability with this methodology; we analyzed the characteristics of this phenomenon by comparing several targeted locations on inflamed vessels. Morphological changes in leukocytes and the intensity of blood flow were also evaluated.

## 2. Materials and Methods

### 2.1. Mice

LysM-GFP mice were used for two-photon intravital imaging [13, 14]. The animal and experimental facilities were located at Yonsei University College of Medicine's specific pathogen-free zone. Only heterogenetic mice (GFP/+) were used for experiments. The institutional review board of Yonsei University College of Medicine approved the study.

### 2.2. Cremaster Exteriorization for Intravital Imaging

The mouse cremaster muscle was chosen for intravital imaging, as it can be locally stimulated by exposure to fMLP after exteriorization [10]. Mice were anesthetized with a Zoletil and Rompun mixture (3 : 2) via intramuscular injection (10 mg/kg). Texas Red dextran was injected via i.v. injection in order to label the blood flow. After a few seconds, mice were placed on the cremaster imaging chamber and firmly fixed with surgical tape. Body hair was removed with a razor and shaving cream along the scrotum and testes. The upper skin of the scrotal sac was retracted with forceps and secured with insect pins. After this procedure, the mouse skin was ready for exposure. From this point, 1x phosphate-buffered saline (PBS) preheated to around 34–35°C was always running inside the chamber sink in order to maintain the temperature and humidity of the cremaster tissue. Attachment of a yellow tip to the PBS tube was useful for continuous water irrigation. Under stereoscopic microscopy, the ventral surface of the pinned scrotal sac was incised. The testicle was removed through the sac with mild pressure on the abdominal cavity. Connective tissues between the scrotal skin and cremaster muscle were gently detached, and the separated scrotal skin was everted to avoid disturbing the cremaster muscle preparation. Finally, the cremaster muscle was gently spread on the silicone bed with several pins. Before imaging, the prepared cremaster muscle was washed once again with preheated 1x PBS in order to obtain a clear view.

### 2.3. Two-Photon Intravital Imaging of Inflamed Cremaster Muscle Venules

After cremaster surgery, vessel inflammation was induced with drops of fMLP (1 *µ*M). Multiphoton microscopy (LSM 7 MP; Zeiss) with two-photon excitation (3 colors: blue, green, and red. Each of the three channels has an emission wavelength of 420–475 nm, 500–550 nm, and 565–610 nm.) was used at a wavelength of 880–900 nm. Second harmonic generation was represented by blue, and Texas Red dextran and neutrophils were visualized with red and green, respectively. MaiTai Deep See laser system (Spectra-Physics) which detects wavelengths from 690 to 1040 nm was used for excitation. Video resolution was fixed at 512 × 512 pixels.

### 2.4. Vascular Permeability Analysis

**Figure 1:**
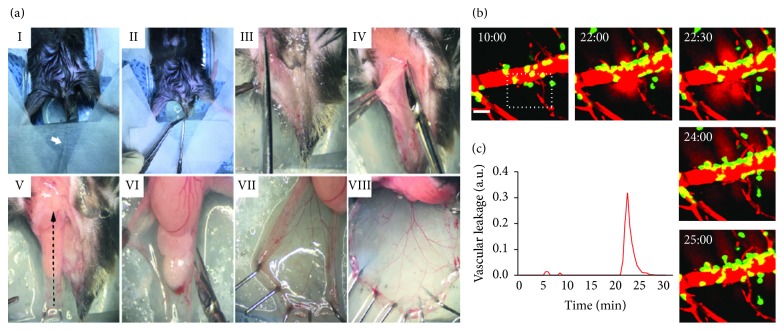
Cremaster surgery and intravital imaging of inflamed cremaster vessel. (a) (I) The body of the mouse was fixed in a chamber, using surgical tape, with traction on the scrotum using suture thread. (II) The scrotum was cut with scissors after filling the chamber with sterile 1x PBS. (III) The epidermis in the scrotum was cut along its edge. (b, c) Changes in vessel permeability with time (min) were observed using intravital imaging. Vascular leakage was measured as an arbitrary unit. An intact vessel with zero leakage was set as the baseline for this unit. A graph of vascular leakage shows local and transient increase in blood permeability. Scale bar: 30 *µ*m. See Video 1.

**Figure 2:**
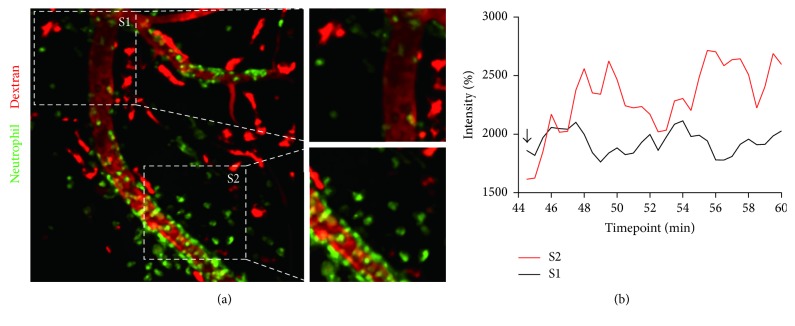
Vascular leakage occurs at specific sites on a vessel despite uniform fMLP treatment. (a) Blood vessels are visualized with dextran, and neutrophils appear green on the image. When fMLP was used to activate neutrophils, the site of leakage was designated as S2 and the area where leakage did not occur was designated as S1 in the same blood vessel (blood leakage occurred at S2, with no apparent change in S1). Scale bar: 30 *µ*m. See Video 2. (b) When leakage occurred at S1 and S2, blood was extruded and the intensity of red was increased. The graphical display compares the intensity at two areas at 44 to 60 min in Video 2. At the time of initial imaging (arrow), the intensity of S1 is higher than that of S2 because the background becomes reddish as overlap occurs due to motion during in vivo imaging. Graph B shows the percent intensity (%) of blood flow in the two areas, as measured by the leakage of dextran.

**Figure 3:**
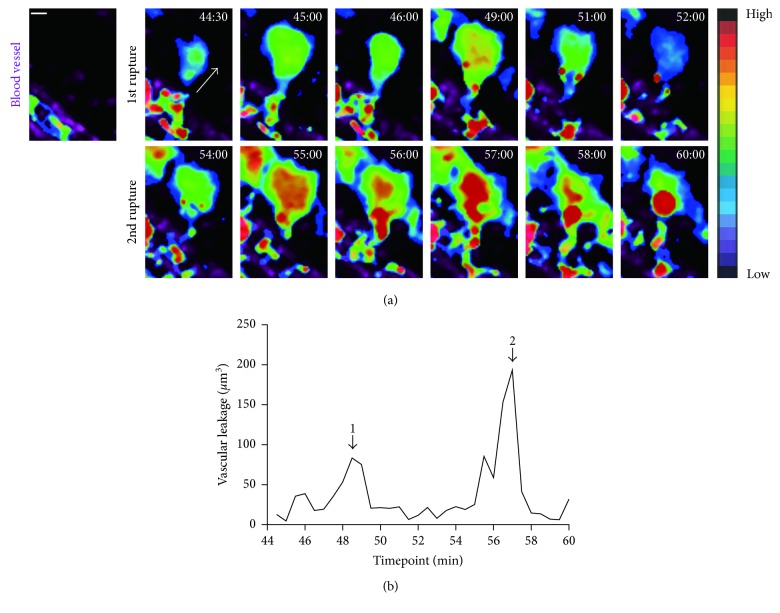
Leakage occurs in a repetitive and consistent manner. (a) The first image without blood leakage in S2 was set as a control condition. Color change shows the amount of leakage. Vessels appear magenta and blood flow is seen with rainbow colors. It is apparent that two disruptive events occurred within 16 min. A much greater amount of leakage was observed during the second disruptive event. (b) Vascular permeability was visually demonstrated, and the graph quantitatively shows about twice as much leakage. The graphical display is consistent with transient leakage from the same site. Endothelial barrier disruption did not always result in leakage. Two arrows in the figure indicate that leakage has occurred. Graph B shows the volume of leakage measured according to site. Scale bar: 10 *µ*m.

Vascular permeability or leakage was measured using an arbitrary unit, a.u. An intact vessel with zero leakage was set as the baseline for this unit (Figure 1). Absolute values were used to represent intensity in % and blood leakage volumes (Figures 2 and 3).

### 2.5. Imaging Data Analysis

ZEN operating software (Carl Zeiss) was used for operating software of two-photon microscope and data gathering. Volocity (PerkinElmer) was used for tracking of neutrophils and vascular permeability. Graphs were drawn using Prism Software (GraphPad).

## 3. Results

### 3.1. Angiorrhexis Is a Local and Transient Event throughout a Blood Vessel during Inflammation

fMLP is a bacterial chemoattractant used to induce an inflammatory state, in which neutrophils infiltrate across blood vessels in damaged or infected interstitium; this may induce vascular disruption [15–17]. The mouse cremaster muscle was prepared for this experiment since a few drops of fMLP can be directly applied to induce vascular inflammation (Figure 1). We hypothesized that fMLP-induced inflammation would lead to neutrophil infiltration and disruption of the vascular barrier in whole blood vessels. However, when fMLP was evenly applied to the exteriorized cremaster muscle, vascular leakage only increased in part of the blood vessel (Figure 1 and Video 1). Thus, disruption did not develop simultaneously in the entire inflamed vessel. On the contrary, vascular leakage occurred locally, and the intensity varied significantly between intact and damaged vessel walls (Figure 1). In addition, neutrophil recruitment was only observed near the site of vessel wall disruption (S2 in Figure 2 and Video 2), and only a few neutrophils were detected near the intact vessel structures (S1 in Figure 2 and Video 2). Interestingly, neutrophils in S2 were attached in a row at the outer surface of the weakened vessel, as if supporting the unstable membrane. Comparison of S1 and S2 clearly showed differences in inflammation progression. Indeed, the change in background intensity, namely, the black space in Figure 2, showed that blood leakage was more intense at S2 than at S1 (Figure 2). Therefore, even though it is known that neutrophil infiltration does not coincide with vascular leakage at the same site in a blood vessel [18, 19], neutrophil adhesion to the abluminal side could induce vascular leakage at a delayed time point following infiltration at the same site. As intravital time-lapse imaging was performed in live animals, shaking occasionally occurred during image capture. This could cause cremaster muscle contraction and negligible variation in Texas Red intensity, as indicated by the arrow in S1 in Figure 2.

### 3.2. Repetitive and Transient Leakage Occurs at a Consistent Point on the Inflamed Blood Vessel

Vascular leakage is a transient and local event in the blood vessel under inflammation. Thus, a point on the stimulated vessel can be more rapidly disrupted than another site on the same stimulated vessel, as shown in Figures 1 and 2. After detection of vascular leakage at a vulnerable point on the inflamed vessel (S2 in Figure 2), we further tracked the target area to quantify blood outflow using a rainbow scale. The principle of the rainbow scale is similar to that of the contour line; when the rate of leakage is high, the area appears red. Areas with relatively low rates of leakage appear blue to green. From the rainbow scale-based tracking of S2, we observed that there were two separate disruptive events at the same site on the vessel (Figure 3). A period of mild vascular leakage was observed at one site, with outflow of about 75 *μ*m^3^ of dextran at peak for 48–50 min. Once outflow began, the Texas Red dextran quickly and widely spread across the interstitial area and became undetectable on two-photon intravital imaging. Interestingly, after an interval of 5 min, another more significant leakage event occurred at the same site on the blood vessel. The peak volume of the second leakage event reached 180 *μ*m^3^ (Figure 3). Based on the graphical display of vascular leakage, blood was suddenly extruded from this particular site on the vessel, and the second extrusion phase was more significant than the first. Thus, leakage from inflamed vasculature is a discontinuous event and may repeatedly occur depending on the progress of inflammation. Therefore, our data verified that two-photon intravital microscopy can be used as a precise tool for research on leakage at an individual vascular level.

## 4. Discussion

Traditional *in vitro* methodologies used to study vascular permeability include measurement of hydraulic conductivity, transendothelial electrical resistance, and albumin transport [20]. However, these techniques are inadequate for replication of actual vascular condition and disease models. In addition to the methods described above, a human microvascular endothelial cell (hMVEC) assay is used to reproduce an actual microvascular environment. With this hMVEC assay system, scientists can directly apply cytokines and growth factors such as interleukin-8 and VEGF to induce inflammatory conditions that are similar to an *in vivo* setting [21]. However, it is important to note that almost every *in vitro* experiment shows greater permeability levels compared to *in vivo* trials [20]. *In vitro* studies are performed under precisely controlled conditions, whereas *in vivo* studies are always affected by complicated and confounding factors.

Vascular permeability assays *in vivo* using Evans blue dye can be useful in overcoming the limitation of cellular and membrane-based experiments. Intravenous injection of Evans blue, a dye that binds to albumin, will circulate throughout the animal body. Due to the impermeability of the endothelium to albumin, Evans blue should remain inside the liver in the control animal [22]. However, when vascular permeability is increased with inflammatory stimulation, leakage of Evans blue will be detected around affected vessels and organs. For example, Han et al. [23] were able to prove that angioedema is mediated by bradykinin via Bk2R by comparing the intensity of Evans blue concentration in the digested mouse ear and intestinal tissues. A vascular permeability assay, however, cannot directly show real-time leakage of blood. Combining our cremaster fluorescence analysis and Evans blue dye assay may compensate for the shortcomings of these methods.

In this study, we performed two-photon intravital microscopy to measure how vascular leakage occurs at the subvascular level under fMLP-induced inflammatory conditions. The cremaster muscle is useful for intravital fluorescent imaging since it can be easily exteriorized and is transparent. We investigated how blood vessels are disrupted and vascular leakage occurs during acute inflammation in correlation with neutrophil infiltration. Therefore, we used LysM-GFP mice, in which neutrophils were clearly visualized by detection of green fluorescence following intravenous injection of Texas Red dextran (70 kDa). Texas Red dextran demonstrates a green color in the absence of bleeding, with use of a long wavelength laser (880–900 nm). This wavelength rarely induces tissue phototoxicity or photobleaching. Thus, it was possible to perform intravital imaging at the same site for up to several hours without severe photo-induced damage. When injected, dextran circulates within and clearly outlines the vasculature. Therefore, Texas Red leakage is a marker of vascular disruption. We locally induced an acute inflammatory state by treatment of the exteriorized mouse cremaster muscle with fMLP. As precise two-photon intravital imaging detects the concentration of Texas Red dextran inside and outside blood vessels, we were able to visualize how leakage occurs from any site on a blood vessel in a living animal. Therefore, this intravital imaging method enables more accurate assessment of vascular leakage in terms of time and location. As shown in our data, vascular leakage is a local and transient event in a blood vessel during acute inflammation. Use of intravital imaging to visualize leakage of intravascular components under inflammatory conditions makes it possible to detect subvascular level disruption of the blood vessel in the target organ. In combination with further research on signaling mechanisms such as cytokine interactions among cells and receptors, this fluorescent intravital approach can provide essential information for development of therapeutic methods for diseases associated with blood vessel malfunction.

## Data Availability

The data used to support the findings of this study are available from the corresponding author upon request.

## Supplementary Materials

Supplementary 1 Video 1: transient and local vascular leakage during CXCL2 stimulated blood vessel.

Supplementary 2 Video 2: spatiotemporal vascular rupture induced vascular permeability increase.
